# A Bacterial Biosensor for Oxidative Stress Using the Constitutively Expressed Redox-Sensitive Protein roGFP2

**DOI:** 10.3390/s100706290

**Published:** 2010-06-24

**Authors:** Carlos R. Arias-Barreiro, Keisuke Okazaki, Apostolos Koutsaftis, Salmaan H. Inayat-Hussain, Akio Tani, Maki Katsuhara, Kazuhide Kimbara, Izumi C. Mori

**Affiliations:** 1 Institute of Plant Science and Resources, Okayama University, Kurashiki 710-0046, Japan; E-Mails: cr_arias@rib.okayama-u.ac.jp (C.R.A.-B.); okazakikeis@gmail.com (K.O.); apostolosk@gmail.com (A.K.); atani@rib.okayama-u.ac.jp (A.T.); kmaki@rib.okayama-u.ac.jp (M.K.); tkkimba@ipc.shizuoka.ac.jp (K.K.); 2 Environmental Risk Management Authority, PO BOX 131, Wellington 6140, New Zealand; 3 Faculty of Allied Health Sciences/UKM Medical Molecular Biology Institute, Universiti Kebangsaan Malaysia, Jalan Raja Muda Abdul Aziz, Kuala Lumpur 50300, Malaysia; E-Mail: salmaan@streamyx.com (S.H.I.-H.); 4 Department of Materials Science and Chemical Engineering, Shizuoka University, Hamamatsu 432-8561, Japan

**Keywords:** oxidative biosensor, redox-sensitive GFP, ratiometric measurement, ROS, environmental stressors

## Abstract

A highly specific, high throughput-amenable bacterial biosensor for chemically induced cellular oxidation was developed using constitutively expressed redox-sensitive green fluorescent protein roGFP2 in *E. coli* (*E. coli*-roGFP2). Disulfide formation between two key cysteine residues of roGFP2 was assessed using a double-wavelength ratiometric approach. This study demonstrates that only a few minutes were required to detect oxidation using *E. coli*-roGFP2, in contrast to conventional bacterial oxidative stress sensors. Cellular oxidation induced by hydrogen peroxide, menadione, sodium selenite, zinc pyrithione, triphenyltin and naphthalene became detectable after 10 seconds and reached the maxima between 80 to 210 seconds, contrary to Cd^2+^, Cu^2+^, Pb^2+^, Zn^2+^ and sodium arsenite, which induced the oxidation maximum immediately. The lowest observable effect concentrations (in ppm) were determined as 1.0 × 10^−7^ (arsenite), 1.0 × 10^−4^ (naphthalene), 1.0 × 10^−4^ (Cu^2+^), 3.8 × 10^−4^ (H_2_O_2_), 1.0 × 10^−3^ (Cd^2+^), 1.0 × 10^−3^ (Zn^2+^), 1.0 × 10^−2^ (menadione), 1.0 (triphenyltin), 1.56 (zinc pyrithione), 3.1 (selenite) and 6.3 (Pb^2+^), respectively. Heavy metal-induced oxidation showed unclear response patterns, whereas concentration-dependent sigmoid curves were observed for other compounds. *In vivo* GSH content and *in vitro* roGFP2 oxidation assays together with *E. coli*-roGFP2 results suggest that roGFP2 is sensitive to redox potential change and thiol modification induced by environmental stressors. Based on redox-sensitive technology, *E. coli*-roGFP2 provides a fast comprehensive detection system for toxicants that induce cellular oxidation.

## 1. Introduction

Physicochemical analysis provides accurate information on the composition of complex environmental samples. Nevertheless, it is widely accepted that this approach by itself fails to provide thorough information on the deleterious effects on living organisms because of its inability to correctly evaluate bioavailability [1]. Biosensor- or bioassay-based toxicity tests intend to complement physicochemical analysis by using biological responses/endpoints to evaluate toxicity related effects. On this note, biosensor technologies for environmental monitoring have been under constant evolution [2].

A wide variety of environmental stressors can alter the intracellular redox status by means of oxidative damage ultimately leading to cell death [3]. The homeostatic imbalance of the redox potential is generated as a consequence of the cell’s inability to cope with the cumulative generation of the reactive oxygen species (ROS): hydrogen peroxide (H_2_O_2_), hydroxyl radical (OH•) and superoxide anion (O_2_•^−^). Among environmental toxicants, metal ions and metalloid oxyanions have been documented to exert their toxic effect through alteration of the cellular redox status [4–7].

On this note, bacteria have been preferably used for the rapid detection of ROS due to their fast response, high growth rate, and low cost [8–12]. Whole-cell bacterial biosensing systems rely extensively on the use of promoter-reporter expression systems, which comprise a transcription regulator plus promoter/operator with an open reading frame for proteins of measurable activity. Exposure to xenobiotics activates the promoter through stress signal transduction events resulting in protein expression. Detectable expression times for these systems range from tens of minutes to hours.

In order to develop more sophisticated oxidative stress biosensors, high levels of specificity, sensitivity, speed, as well as high-throughput amenability have become desirable features. In this respect, since the design by Hanson *et al.* [13], several studies have profited from redox-sensitive green fluorescent proteins (roGFPs), for the live redox monitoring of intracellular oxidation state, *c.f.,* yeast [14,15], plants [16,17] and mammalian cells [18,19]. These GFP mutants allow ratiometrical quantification of the redox potential based on the formation of disulfide bonds between surface-exposed cysteine residues. Fluorescence patterns of excitation, which represent oxidized and reduced forms of the protein denote the extent of oxidation. In terms of analytical advantage, errors resulting from variations of the indicator concentration, photobleaching and variable cell thickness are reduced or eliminated [18]. Furthermore, when compared to the promoter-reporter approach, the measurement benefits from a reduction of the analysis time because it is based on the conformational changing velocity of a constitutively expressed protein, as opposed to the time required for its expression.

Despite its beneficial characteristics, an integrating approach for live redox monitoring of chemically induced oxidative stress in bacterial cells has not yet been undertaken. In the present study, we developed a ratiometric fluorescence assay using roGFP2 (GFP mutant C48S/S147C/Q204C/S65T/Q80R) [13] expressed in *Escherichia coli*, to monitor chemically induced oxidative stress. The redox-sensitive properties of the roGFPs provided the basis for the engineering of a bioassay for cellular oxidation. Furthermore, the experiments are shown to be high-throughput amenable, to acquire data on a scale of seconds to minutes.

## 2. Results

### 2.1. Autofluorescence normalization and background oxidation

**Appendix Figure 1.**
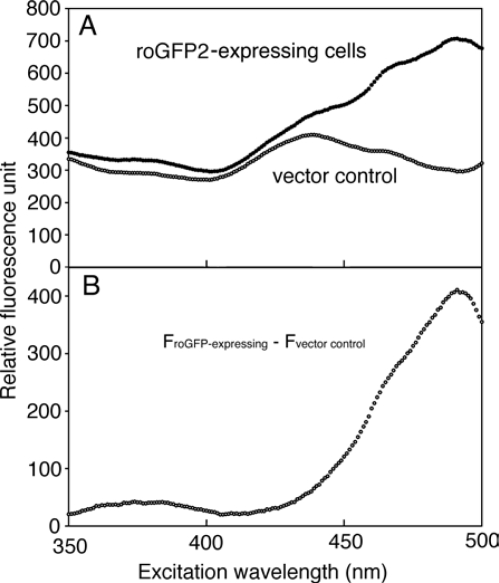
Fluorescence of roGFP2 expressed in *E.coli* cells. (A) fluorescence spectra of roGFP2-expressing cells (filled circle) and vector control (open circle), (B) subtraction of vector control from the roGFP2-expressing spectrum. Each data point was obtained within a 1 nm interval. The data presented shows the results from a single typical experiment of 3 replicates.

Fluorescence was detectable in roGFP2-transformed *E. coli* cells and non-transformants. Compared fluorescence spectra of roGFP2-expressing cells and the vector control (autofluorescence) are shown in Appendix Figure 1A Subtraction of vector-control fluorescence generated the distinctive roGFP2 spectrum (Appendix Figure 1B) in accordance with previous data of purified roGFP2 protein [13].

**Figure 1.**
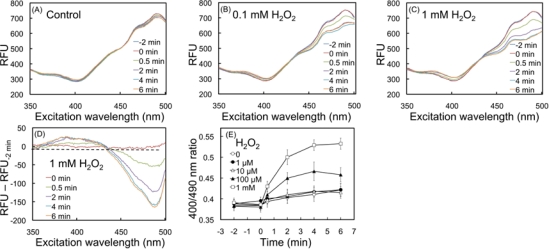
Spectroscopic analysis of *E. coli*-roGFP2 after H_2_O_2_-induced cellular oxidation. Fluorescent excitation spectra of a typical kinetic experiment starting at −2 minutes (before the addition of H_2_O_2_), 0 minute (addition), up to 6 minutes after addition of the chemical with water (A), 0.1 mM H_2_O_2_ (B) and 1 mM H_2_O_2_ (C). Clarified spectral behavior after treatment with 1 mM H_2_O_2_ and subsequent subtraction of basal fluorescence at −2 minutes (D). Kinetic analysis of the 400/490 nm ratiometric response (E) (open squares: 1 mM, closed triangles: 0.1 mM, open triangles 10 mM, closed circles: 1 mM, and open circles: water control). Error bars show ±S.E.M.

**Appendix Figure 2.**
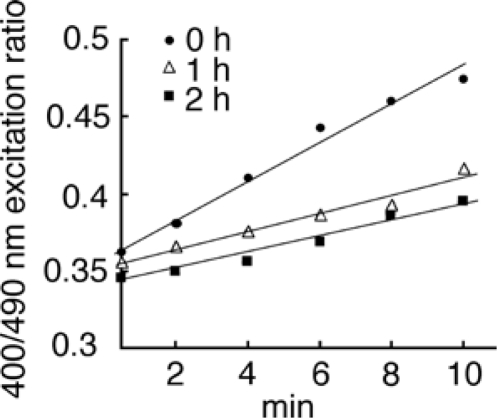
Auto-oxidation measurement of roGFP2 in *E. coli* cells and time dependent relief from turbulence. Fluorescence was measured spectrophotometrically, immediately after preparing the cell suspension (0 h) and after settling the cells for 1 h (1 h) and 2 h (2 h).

Shortly after preparation of the cell suspension, increasing spontaneous cellular oxidation was observed in the absence of toxicants (Appendix Figure 2, 0 h). However, standing for 1 hour at room temperature reduced the spontaneous oxidation rate (Appendix Figure 2, 1 h). Further stagnation resulted in a slight reduction of the rate (Appendix Figure 2, 2 h). The spontaneous oxidation was sometimes not clearly observed in multi-well plate reader experiments as seen in Figure 3. This might be due to difference in shaking procedures: continuous stirring for spectroscopy (Appendix Figure 2 and Figure 1E), and intermittent shaking for multi-well plate analysis (Figure 3).

### 2.2. Response of the biosensor to H_2_O_2_

Hydrogen peroxide is known to evoke oxidation in prokaryotic and eukaryotic cells. roGFP2 has been found to successfully indicate cell oxidation by H_2_O_2_ in mammalian [13] and plant cells [16]. Here, *E. coli* cells constitutively expressing roGFP2 (*E. coli*-roGFP2) were challenged with H_2_O_2_ to examine spectral and ratiometric fluorescence brought about by cellular redox changes.

Figure 1 shows the spectral response of the biosensor to H_2_O_2_. Compared to the control (Figure 1A), 0.1 and 1 mM of H_2_O_2_ induced spectral pattern changes within 6 minutes (Figure 1B and C). Fluorescence spectra displayed two distinctive excitation maxima at 400 (F_ex400_) and 490 nm (F_ex490_) related to its oxidized and reduced form, respectively. A decrease of F_ex490_ induced by H_2_O_2_ became apparent at 30 seconds and progressed further in time (>2 minutes) (Figure 1B and C). Also, an increase of F_ex400_ was observed, although the magnitude of the change was smaller than that of F_ex490_. Subtraction of the basal spectrum at t = −2 minutes (before H_2_O_2_ addition) from the other spectra showed that the F_ex400_ increase and F_ex490_ decrease (Figure 1D), providing evidence that fluorescence change unequivocally indicated oxidation of the biosensor.

Figure 1E shows increasing F_ex400_/F_ex490_ ratio at high H_2_O_2_ concentrations. An oxidation maximum at 100 μM H_2_O_2_ was reached in 4 minutes (Figure 1E), while approximately a period of 15 minutes was necessary for mammalian cells to reach the same state [18]. These results demonstrate the capability of roGFP2 to monitor oxidation in *E. coli* cells, as previously reported for mammalian [18] and plant cells [16].

### 2.3. Spectral changes of the biosensor upon exposure to toxic metal compounds

Monitoring of cell oxidation was carried out after exposure of *E. coli*-roGFP2 to the environmentally relevant heavy metal (HM) cations Cd^2+^, Cu^2+^, Pb^2+^ and ^+^ Zn^2+^; and the oxyanions AsO_2_^−^ and SeO_3_^2−^.

**Figure 2.**
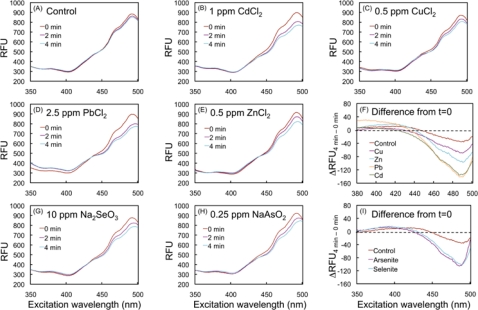
Spectroscopic analysis of *E. coli*-roGFP2 after induction of cellular oxidation by heavy metal compounds and oxyanions. Fluorescent excitation spectra of kinetic experiments after exposure of 4 minutes to water (A), 1 ppm CdCl_2_ (B), 0.5 ppm CuCl_2_ (C), 2.5 ppm PbCl_2_ (D) and 0.5 ppm ZnCl_2_ (E). Overall comparison among control and cations at 4 minutes after subtraction from 0 minute (E). Kinetic data obtained after 4 minutes exposure to 0.25 ppm NaAsO_2_ (F) and 10 ppm Na_2_SeO_3_ (G). Overall comparison among oxyanions at 4 minutes after subtraction from 0 minute (I).

Figure 2 shows the effect of HM cations on the fluorescence of the biosensor. Fluorescence changes, indicating oxidation of the biosensor, were observed at 4 minutes by addition 0.5 ppm Cu^2+^, 0.5 ppm Zn^2+^, 1 ppm Cd^2+^ and 2.5 ppm Pb^2+^ (Figure 2B-E). Ten ppm SeO_3_^2−^ and 0.25 ppm AsO_2_^−^ showed a comparable response to HM cations upon exposure (Figure 2G and H). Increase of F_ex400_ and decrease of F_ex490_ were clearly observed, when subtracted from the t = 0 minute data, demonstrating oxidation of roGFP2 (Figures 2F and I). The data provide evidence on the capabilities of the biosensor to detect the HM and metalloid-evoked cell oxidation.

### 2.4. Ratiometric characterization of the biosensor

**Figure 3.**
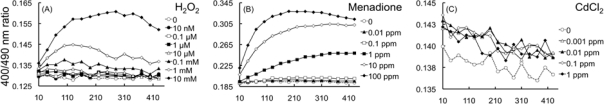

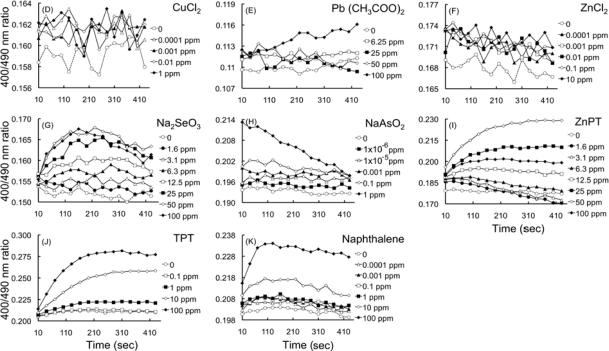
Multi-well ratiometric kinetic analysis of cellular oxidation using *E. coli*-roGFP2. Exposure to several concentrations of H_2_O_2_ (A), menadione (B), CdCl_2_ (C), CuCl_2_ (D), Pb(CH_3_COO)_2_ (E), ZnCl_2_ (F), Na_2_SeO_3_ (G), NaAsO_2_ (H), ZnPT (I), TPT (J) and naphthalene (K). Each data point represents the average of six replicates. Measurements were made every 30 seconds during 7 minutes. Results shown correspond to a typical experiment, where each data point represents the quotient of the excitation fluorescence relative value at 400 and 490 nm.

Kinetic analyses were performed with a multiwell plate reader to further characterize oxidation patterns of the *E. coli*-roGFP2 biosensor in a high throughput fashion. F_ex400_ and F_ex490_ were measured every 30 seconds during 7 minutes (Figure 3). The first data point was obtained at 10 seconds after the addition of chemicals due to the measurement lag time.

#### 2.4.1. Detection of oxidation upon exposure to H_2_O_2_ and menadione

**Figure 4.**
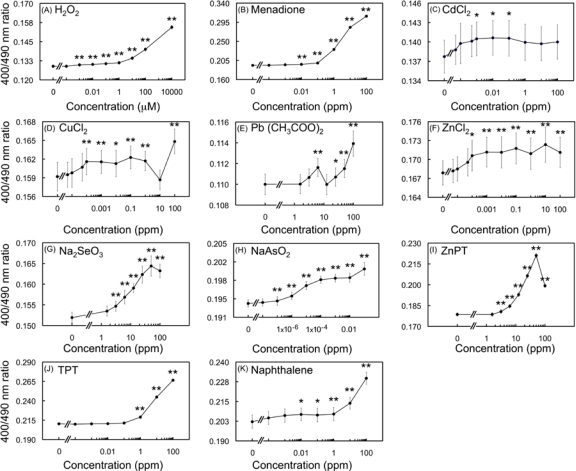
Concentration-response curves as shown by fluorescent ratiometric changes. Exposure to several concentrations of H_2_O_2_ (A), menadione (B), CdCl_2_ (C), CuCl_2_ (D), Pb(CH_3_COO)_2_ (E), ZnCl_2_ (F), Na_2_SeO_3_ (G), NaAsO_2_ (H), ZnPT (I), TPT (J) and naphthalene (K). Each data point represents the ratiometric average of the dataset during 7 minutes (10–430 seconds) for a given concentration. Significant differences between control (concentration=0) and treated groups are indicated by * (*p* < 0.05) and ** (*p* < 0.01) using Dunnett’s test followed by ANOVA. Error bars show ±S.E.M. Error bars in panels A, B, I and J are too small to be seen.

A gradual increase of the F_ex400_/F_ex490_ ratio was observed with 1 and 10 mM H_2_O_2_ reaching the plateau within 100–200 seconds (Figure 3A). In order to assess concentration dependency of biosensor oxidation, the average of F_ex400_/F_ex490_ ratio from 10–430 seconds was plotted against concentrations of added chemicals (Figure 4). The concentration-dependency curve showed a gradual increase proportionally to H_2_O_2_ concentration, and the lowest observed effective concentration (LOEC) was determined as low as 10 nM (*p* < 0.01) (Figure 4A).

Figure 3B shows the maximum oxidation was reached at approximately 200 seconds after exposure to menadione (0.1–100 ppm). The concentration-response correlation demonstrated the LOEC to be 0.01 ppm at *p* < 0.01 (Figure 4B). Conclusively, sigmoid response curves were observed for both H_2_O_2_ and menadione treatments (Figures 4A and B) further establishing oxidation causality under increasing concentrations of the chemicals.

#### 2.4.2. Effects of HM cations on *E. coli*-roGFP2

Treatments with Cd^2+^, Cu^2+^, Pb^2+^ and Zn^2+^ showed an irregular kinetic behaviour together with positive ratiometric responses (Figure 3C-F), unlike H_2_O_2_ and menadione, which increased gradually over time. Moreover, ratiometric changes were smaller compared to H_2_O_2_ and menadione. An exception was observed, however, at 100 ppm of Pb(CH_3_COO)_2_ where an almost linear increase was triggered. Although significant differences with respect to the controls were found, concentration-dependent behaviour did not show typical sigmoid curves in any of the treatments but rather fluctuating ones, following immediate oxidation after exposure to HMs (Figure 4C-F). This behaviour may be explained by the coordination to side chains of cysteine residues and cross-link of thiol groups with HM cations due to their thiophilic nature [20]. The affinity to Cd^2+^, Cu^2+^ and Pb^2+^, and in a lesser degree Zn^2+^, alters the conformation of roGFP2. Although initial exposure to metal cations elicit the production of ROS, it is possible that the formation of roGFP-sulfide complexes can inhibit the structural changes that account for the F_ex400_/F_ex490_ ratiometric response.

**Figure 5.**
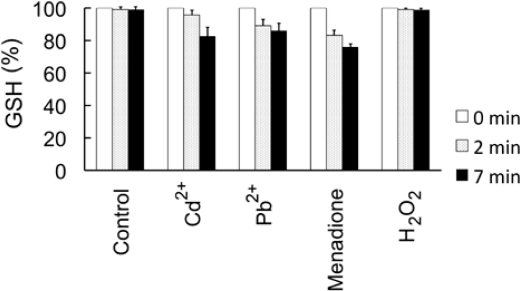
Effect of Cd^2+^, Pb^2+^, H_2_O_2_ and menadione on intracellular levels of GSH. Changes in concentration of GSH as quantified as described in Materials and methods are shown at 2 and 7 minutes after exposure to 0.1 ppm CdCl_2_, 1 ppm Pb(CH_3_COO)_2_, 1 ppm menadione and 1 mM H_2_O_2_. Control corresponds to cells treated with H_2_O. Six replicates were used for each dataset. Error bars indicate standard deviation.

To evaluate the involvement of HMs and sulfhydryl-rich groups, we next determined the content of reduced glutathione (GSH) in *E. coli*-roGFP2 cells under representative concentrations of HMs (Figure 5). The exposure induced reduction of GSH contents by 4.0 and 16.8% at 2 minutes, and 10.6 and 14.2% at 7 minutes, for Cd^2+^ and Pb^2+^ respectively. Interestingly, H_2_O_2_ did not induce a significant reduction of GSH (0.9 and 1.1%). Control (H_2_O) values were kept below 1% for both timepoints. Menadione showed essentially the same result as the metals, with 16.8 and 24.0% reduction at 2 and 7 minutes, respectively (Figure 5). Hydrogen peroxide and menadione effects were in accordance with previous data obtained in *E. coli* by Smirnova *et al*. [21].

**Figure 6.**
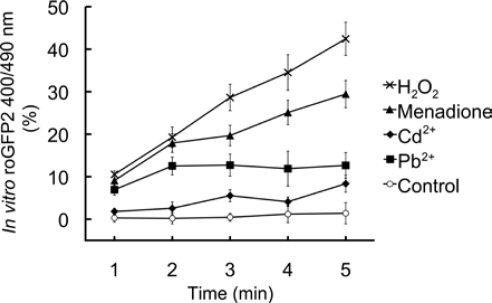
Effect of Cd^2+^, Pb^2+^, H_2_O_2_ and menadione and on roGFP2 *in vitro*. A 1.39 μg/mL solution of roGFP2 was exposed to 0.1 ppm CdCl_2_, 1 ppm Pb(CH_3_COO)_2_, 1 ppm menadione and 1 mM H_2_O_2_. Changes in 400/490 nm fluorescence ratio were monitored for 5 minutes with a multi-well plate reader and expressed in percentage compared to the value at t = 0 minute. Three replicates were used for each dataset and error bars indicate standard deviation.

Additionally, ratiometric changes of isolated roGFP2 *in vitro* were determined to observe the direct interaction of roGFP2 protein and chemicals (Figure 6).

A steady increase was observed for 0.1 ppm Cd^2+^ and 1 ppm Pb^2+^ ranging from 1.8 to 8.3% and 6.9 to 12.6%, respectively. These increments were smaller than those observed with 1-ppm menadione and 1-mM H_2_O_2_ treatments at 9.1 to 29.4% and 10.5 to 42.4%, respectively. Moreover, a net reduction of the fluorescence signal was not observed while ratiometric response increased (data not shown).

The reduced effects on the direct oxidation of roGFP2 by HMs, when compared to H_2_O_2_ and menadione, may be accounted by the coordination bond formation between HMs and key cysteine residues (C147 and C204) near the chromophore.

Formation of the disulfide bond occurs between both residues deriving in the characteristic fluorescence shift when redox potential is altered. Reduction of GSH, coupled with production of ROS would favour an oxidizing environment while initial sensitivity and affinity of roGFP2 would be hindered resulting in the irregular kinetic and concentration-dependent patterns. These counteracting effects might also explain the large variations observed in Figure 4C–F. Despite the lack of regular response patterns, the HM data can be differentiated from responses of other oxidative chemicals, based on their particular ratiometric patterns. Notwithstanding the anomalous responses, LOECs could be determined at 1 × 10^−3^, 1 × 10^−4^, 6.3 and 1 × 10^−3^ ppm at *p* < 0.05 for Cd^2+^, Cu^2+^, Pb^2+^ and Zn^2+^, respectively (Figure 4).

#### 2.4.3. Oxidation detected upon exposure to selenite and arsenite

Selenite and arsenite are environmentally relevant pollutants and known to induce oxidative stress as a mechanism of their toxicity. Thus, we next examined arsenite and selenite-induced cell oxidation using the *E. coli*-roGFP2 biosensor.

Exposure of the biosensor to SeO_3_^2−^ induced a gradual increase of the F_ex400_/F_ex490_ ratio within 150–200 seconds at 12.5–100 ppm (Figure 3G), contrary to HM that displayed irregular kinetics. Response did not increase at concentrations higher than 50 ppm SeO_3_^2−^ (Figure 4G).

In arsenite-induced oxidation, an immediate rise of the F_ex400_/F_ex490_ ratio occurred within 10 seconds, followed by a progressive reduction when 1 ppm AsO_2_^−^ was added (Figure 3H). Notably, intensity of F_ex490_ was reduced gradually by the same concentration suggesting that the ratiometric reduction is most likely due to its strong toxic effects. For lower concentrations (1 × 10^−6^−0.1 ppm) maximum responses were reached within the first 100 seconds.

Both SeO_3_^2−^ and AsO_2_^−^ treatments showed a concentration dependent response (Figures 4G and H) with a great difference in their sensitivity, *i.e.,* the LOECs for SeO_3_^2−^ and AsO_2_^−^ were determined as low as 3.1 ppm (*p* < 0.05) and 1x10^−7^ ppm (*p* < 0.01), respectively (Figures 4G and H).

#### 2.4.4. Detection of oxidation caused by organometallic compounds

Figures 3I and 3J show the results of oxidation of the biosensor by two organometallic compounds which are used as biocides, zinc pyrithione (ZnPT) and triphenyltin (TPT), respectively. Exposure of cells to ZnPT generated gradual increases in the fluorescence ratios (12.5–100 ppm). Similar gradual time-dependent increases were observed for TPT at 1, 10 and 100 ppm (Figure 3J). Both compounds reached a maximum level of oxidation between 250 and 310 seconds after the exposure. Furthermore, both compounds significantly induced cell oxidation with similar LOECs of 1.56 ppm at *p* < 0.01 and 1 ppm at *p* < 0.01 for ZnPT and TPT exposures, respectively (Figures 4I and 4J).

#### 2.4.5. Detection of oxidation after exposure to naphthalene

To further characterize the biosensor, cells were exposed to naphthalene, a two-ring hydrocarbon. Figure 3K shows that a maximum oxidation level was reached within 100 seconds of exposure with 10 and 100 ppm. The LOEC was found to be 1 × 10^−4^ ppm at *p* < 0.05 (Figure 4K). The fact that the biosensor responded to naphthalene indicates versatility for detecting highly lipophilic chemicals, such as poly aromatic hydrocarbons (PAHs).

## 3. Discussion

In this study, chemically induced oxidation of *E. coli* was successfully assessed using roGFP2. Fluorescent ratiometric changes, which denote interchange between oxidized and reduced forms of roGFP2, allow a nearly live measurement of the intracellular redox conditions. Moreover, data showed that roGFP2 expressed in *E. coli* was subject to changes brought about by chemically induced alteration of the intracellular redox status.

Measuring fluorescence of the constitutively expressed protein in cells allowed a very rapid evaluation, reaching maxima within 2 to 6 minutes for most chemicals tested. This high-speed characteristic of the biosensor may allow the analysis of a large number of samples in a short time frame and facilitate time-consuming processes, such as the identification of toxicants.

Genes belonging to regulons SoxRS (induced by O_2_•^−^) [11], OxyRS (induced by H_2_O_2_ and OH•) [8,10] or both [9,12] fused to the *luxCDABE* operon have provided the fusions of choice for the development of oxidative stress biosensors. Note that specificity of regulons to ROS is different. Comprehensive oxidative biosensors should be able to detect the activity of chemicals inducing both oxidative stress regulons. In this study, detection of cellular oxidation was not based on the induction of gene expression, but through the increase of the intracellular redox potential. The *E. coli*-roGFP2 biosensor detected both H_2_O_2_- and menadione-induced oxidation, indicating broad specificity of the biosensor for ROS. Differences between H_2_O_2_ and menadione may be attributed to the ROS produced by the latter, which includes, O_2_•^−^ and H_2_O_2_ [21].

Thiophilic activity may also be corroborated by the speed of the oxidative reaction induced by HMs. Transition divalent cations react immediately with GSH to form bisglutathionate complexes [22]. The presence of cysteine residues should onset a similar reaction velocity in roGFP2, prompting the alteration of its fluorescent properties. A disturbance in protein activity due to HMs is also observed in enzymes containing sulfhydryl groups in their active sites [23]. Although Pb is not a transition metal, it induces oxidative stress through interaction with GSH [5], which might account for the observed effects.

Different kinetic patterns of biosensor oxidation were observed (Figure 3), indicating that oxidation is influenced by the nature of the utilized xenobiotics. The heavy metals Cd^2+^, Cu^2+^, Pb^2+^ and Zn^2+^ only showed 1.6, 3.4, 3.4 and 1.9% oxidation increases, respectively; which were apparently lower than others *i.e.*, (in %) H_2_O_2_, 16.5; menadione, 37.4; SeO_3_^2−^, 7.6; AsO_2_^−^, 5.9; ZnPT, 19.2; TPT, 21.1 and naphthalene, 12 (Figure 3). Soft Lewis acids such as Cd^2+^ and Pb^2+^, and in a lesser degree intermediates Cu^2+^ and Zn^2+^, display a marked preference for coordination to thiol compounds, such as cysteine and GSH [20]. After reaction with GSH, the formed bisglutathionate complex can then react with molecular oxygen rendering the metal cation, H_2_O_2_ and oxidized bisglutahione (GSSG). The latter is in turn reduced again in a NADPH-dependent reaction, and metal cations immediately bind another two glutathione molecules, producing oxidative stress [24].

Approximately 90% of the reduced thiol concentration in *E. coli* corresponds to GSH [25]. It is thus reasonable to consider GSH as the main target of HMs tested. After exposure, an increase in the GSSG/GSH ratio would alter the redox potential and ultimately cause stress. However, disulfide formation between C147 and C20 of roGFP2 would also be hindered. The GSSG/GSH ratio, would thus fail to show the true extent of change of the redox equilibrium as suggested by the anomalous kinetics and non-sigmoid concentration dependency patterns observed for Cd^2+^, Pb^2+^, Cu^2+^ and Zn^2+^.

Recently, roGFP1-R12 was expressed in *Saccharomyces cerevisiae,* and oxidative stress was monitored after exposure to H_2_O_2_, NaAsO_2_ and Pb(NO_3_)_2_ [14]. The authors were, however, unable to perform ratiometric analysis for unclear reasons. The lack of a ratiometric feature would be of critical importance to evaluate the extent of oxidation in roGFP-expressing cells.

*E. coli*-roGFP2 represented bioavailable fractions of a wide variety of environmental oxidants. Interestingly, detection limits for inorganic compounds like AsO_2_^−^ fall within environmentally relevant concentrations [26]. Among organic chemicals, menadione, a naphthoquinone used as an alternative biocide substituting for other nonselective oxidants [27], was detected at the ppb level. Naphthalene, a toxicologically and environmentally relevant PAH, capable of inducing ROS production and oxidative stress [28] also evoked cellular oxidation. Naphthalene is metabolized in mammalian cells to naphthoquinone indicating that a similar process might be involved in bacteria, as the toxicity of naphthoquinones is related to disturbances in intracellular GSH concentrations [29].

Zinc pyrithione is known for its antifouling properties, in addition to its bactericidal and fungicidal activities in cosmetic products [30]. Although reports on the toxicity of ZnPT on various organisms are relatively abundant [31, and references therein], the mechanism of action remains unclear. Here, we showed the redox changes caused by ZnPT in *E. coli* that may provide a probable model for Gram-negative bacteria. This information is of particular importance for ecotoxicological implications. Triphenyl tin, a biocide and pesticide organotin [32], also induced oxidation. This finding is the first report for this mode of action in bacteria according to our best knowledge, and is supported by modification of critical thiol groups in mitochondria leading to cell death [33,34]. It could be speculated that organometallic biocides commonly induce cellular oxidation in Gram-negative bacteria. However, a more thorough examination is required.

A series of results presented in the study suggest a possible application of *E. coli*-roGFP2 biosensor to ecotoxicological evaluation of a wide variety of hazardous chemicals. Further testing should determine the correlation between toxicity and chemical-induced oxidation. Finally, complementing *E. coli*-roGFP2 data with Toxicity Identification Evaluation could provide a valuable tool in the determination of the chemical sources of oxidative stress in environmental samples.

## 4. Materials and Methods

### 4.1. Chemicals

Tested compounds comprised sodium arsenite, cadmium chloride and copper (II) chloride (Wako Pure Chemicals Industries, Osaka, Japan), zinc chloride, lead (II) chloride and lead (II) acetate (Nacalai Tesque, Inc, Kyoto, Japan), menadione (2-methyl-1,4-naphtoquinone) (Mitsuwa Chemical Co., Ltd, Japan), naphthalene (Sigma Aldrich, Tokyo, Japan), disodium selenite and the 30% hydrogen peroxide (Santoku Chemical Industries Co., Ltd., Tokyo, Japan), zinc pyrithione (ZnPT) (Sigma Aldrich, St. Louis, USA) and triphenyltin (TPT) (Fluka, Hong Kong). All chemicals were of the highest purity available. Stocks were prepared using Milli-Q water (Nihon Millipore KK, Tokyo, Japan) or analytical grade dimethyl sulfoxide (DMSO) from Sigma-Aldrich. After preliminary tests, we tested a range of concentrations from non-observable effect concentrations up to 100 ppm, unless otherwise stated.

### 4.2. Bacterial strain

*Escherichia coli* strain DH5α™ (Invitrogen, Tokyo, Japan) cells were transformed with plasmid pRSET-roGFP2 [13], which allows constitutive expression of roGFP2 protein. We chose roGFP2 because of its higher sensitivity over other roGFP variants [13,18]. Ampicillin (50 μg L^−1^) was used to assure plasmid maintenance on Luria-Bertani agar and liquid media.

### 4.3. Spectrophotometric measurement of roGFP2 fluorescence

Five hundred μL of overnight-precultured *E. coli* cells harboring pRSET-roGFP2 (*E. coli*-roGFP2) were inoculated in 50-mL LB liquid medium and further cultivated at 37 °C until optical density at 600 nm reached 1.3. Cells were washed twice in 5 mM 4-(2-hydroxyethyl)-1-piperazineethanesulfonic acid (HEPES) buffer containing 171 mM NaCl (pH 7.0). Resuspended cells in 10 mL of the suspension buffer were left to settle at 20 °C for 1 hour prior testing.

Intensity of fluorescence was measured spectrophotometrically (RF-5300PC, Shimadzu, Tokyo, Japan). Emission wavelength was fixed at 525 nm and excitation wavelength was scanned from 350 to 500 nm. Bandwidths of excitation and emission were set at 3 and 10 nm, respectively. Cell suspension in the cuvette was continuously stirred with a small magnetic bar and an add-on magnetic stirrer. The fluorescence excitation ratios (400/490 nm) were used as index for cellular oxidation [18].

### 4.4. Microplate-based measurement of roGFP2 fluorescence

Cell suspension was prepared identically as described above for the spectrophotometric measurements. Aliquots (99 μL) of cell suspension were placed in the wells of a black flat bottom 96-well assay plates (BD Falcon, New Jersey, USA), followed by settling the cell suspension for 1 hour. One microliter of the selected chemical solution was added in the wells before the beginning of the measurement.

Fluorescence intensity was recorded every 30 seconds using excitation filters 400 and 490 nm (10 and 20 nm bandwidth, respectively). A 528 nm emission filter (20 nm bandwidth) was accordingly used. Measurements were performed in a multi-detection microplate reader (Powerscan HT, Dainippon Sumitomo Pharma, Osaka, Japan).

### 4.5. Measurement of cellular GSH

The content of reduced glutathione (GSH) in *E. coli*-roGFP2 cells was determined according to Ellman [35] with slight modifications as described by Inayat-Hussain *et al*. [36]. Cells were prepared as described above in the ice-cold suspension buffer. Aliquots of 990 μL were distributed in 1.5 mL tubes and kept on ice until used. Ten microliters of the desired chemical solution were added to the tubes followed by incubation at 37 °C. At the desired time (0, 2 and 7 minutes), cells were centrifuged for 5 minutes (13,000 rpm) at 4 °C. Subsequently, pelleted cells were permealized by incubation in the lysis buffer containing 50 mM K_2_HPO_4_, 1 mM EDTA, and 0.1% v/v Triton X-100 (pH 6.5) on ice for 15 minutes. The crude lysates were cleared by centrifugation at 13,000 rpm for 15 minutes at 4 °C. Then, 50 μL of supernatant were mixed with 50 μL solution of 80 mM Na_2_HPO_4_, 0.8 mM EDTA, pH 8.0, and 0.8 mg/mL 5,5′-dithiobis-2-nitrobenzoic acid followed by a 15-minutes incubation at 37 °C. Absorbance at 405 nm was measured using a microplate reader. The concentration of free thiols in the samples was calculated based on reduced GSH as the standard.

### 4.6. In vitro effects of chemicals on roGFP2

After culturing *E. coli*-roGFP2 as mentioned in Section 2.2, cells were resuspended in a buffer containing 50 mM HEPES (pH 7.9), 300 mM NaCl and 10% glycerol, followed by sonication on ice for 7 minutes (VC505, Sonics & Materials, Inc., Newton, CT, USA). After discarding the supernatant, roGFP2 protein, which has a His tag, was purified and concentrated using a Ni^2+^ -nitriloacetic acid-agarose resin (Qiagen, Hilden, Germany) essentially as previously described by Hanson *et al*. (2004). The isolated roGFP2 was fully oxidized. In order to reduce roGFP2, dithiothreitol (DTT) was added to a final concentration of 1 mM, mixed and incubated for 5 minutes. The reduced roGFP2 was subsequently subjected to gel filtration (Sephadex G-25 column, Pharmacia) to replace the buffer to 75 mM HEPES (pH 7.0) and 140 mM NaCl, and remove the remaining DTT. Protein concentration was 1.39 μg mL^−1^, and fluorescence was measured as described in Section 4.3.

### 4.7. Data analysis

In order to determine the lowest observed effect concentrations (LOECs), datasets were compared by one-way ANOVA followed by Dunnett multiple comparison post hoc test. For spectrophotometric measurements, presented data points are the mean of 5 measurements. On the other hand, time points of the 96-well assay data represent the mean of 6 measurements, unless otherwise stated.

## References

[1] Magrisso S, Erel Y, Belkin S (2008). Microbial reporters of metal bioavailability. Microb. Biotechnol.

[2] Farré M, Rodríguez-Mozaz S, López de Alda MJ, Barceló D, Hansen PD, Barceló D, Hansen PD (2009). The Handbook of Environmental Chemistry.

[3] Franco R, Sánchez-Olea R, Reyes-Reyes EM, Panayiotidis MI (2009). Environmental toxicity, oxidative stress and apoptosis; Ménage à trois. Mutat. Res. Genet. Toxicol. Environ. Mutagen.

[4] Shen H, Yang C, Liu J, Ong C (2000). Dual role of glutathione in selenite-induced oxidative stress and apoptosis in human hepatoma cells. Free Radic. Biol. Med.

[5] Ercal N, Gurer-Orhan H, Aykin-Burns N (2001). Toxic metals and oxidative stress part I: Mechanisms involved in metal-induced oxidative damage. Curr. Top. Med. Chem.

[6] Valko M, Morris H, Cronin MT (2005). Metals, toxicity and oxidative stress. Curr. Med. Chem.

[7] Kumagai Y, Sumi D (2007). Arsenic: Signal transduction, transcription factor, and biotransformation involved in cellular response and toxicity. Annu. Rev. Pharmacol. Toxicol.

[8] Belkin S, Smulski DR, Vollmer AC, Van Dyk TK, LaRossa RA (1996). Oxidative stress detection with *Escherichia coli* harboring a katG′::lux fusion. Appl. Environ. Microbiol.

[9] Lee HJ, Gu MB (2003). Construction of a sodA::luxCDABE fusion *Escherichia coli*: Comparison with a katG fusion strain through their responses to oxidative stresses. Appl. Microbiol. Biotechnol.

[10] Mitchell RJ, Gu MB (2004). Construction and characterization of novel dual stress-responsive bacterial biosensors. Biosens. Bioelectron.

[11] Kim BC, Youn CH, Ahn JM, Gu MB (2005). Screening of target-specific stress-responsive genes for the development of cell-based biosensors using a DNA microarray. Anal. Chem.

[12] Niazi JH, Kim BC, Ahn JM, Gu MB (2008). A novel bioluminescent bacterial biosensor using the highly specific oxidative stress-inducible pgi gene. Biosens. Bioelectron.

[13] Hanson GT, Aggeler R, Oglesbee D, Cannon M, Capaldi RA, Tsien RY, Remington SJ (2004). Investigating mitochondrial redox potential with redox-sensitive green fluorescent protein indicators. J. Biol. Chem.

[14] Yu S, Qin W, Zhuang G, Zhang X, Chen G, Liu W (2009). Monitoring oxidative stress and DNA damage induced by heavy metals in yeast expressing a redox-sensitive green fluorescent protein. Curr. Microbiol.

[15] Delic M, Mattanovich D, Gasser B (2010). Monitoring intracellular redox conditions in the endoplasmic reticulum of living yeasts. FEMS Microbiol. Lett.

[16] Meyer AJ, Brach T, Marty L, Kreye S, Rouhier N, Jacquot JP, Hell R (2007). Redox-sensitive GFP in Arabidopsis thaliana is a quantitative biosensor for the redox potential of the cellular glutathione redox buffer. Plant J.

[17] Jubany-Mari T, Alegre-Battle L, Jiang K, Feldman LJ (2010). Use of a redox-sensing GFP (c-roGFP1) for real-time monitoring of cytosol redox status in Arabidopsis thaliana water-stressed plants. FEBS Lett.

[18] Dooley CT, Dore TM, Hanson GT, Jackson WC, Remington SJ, Tsien RY (2004). Imaging dynamic redox changes in mammalian cells with green fluorescent protein indicators. J. Biol. Chem.

[19] Gutscher M, Pauleau AL, Marty L, Brach T, Wabnitz GH, Samstag Y, Meyer AJ, Dick TP (2008). Real-time imaging of the intracellular glutathione redox potential. Nat. Methods.

[20] Hobman JL, Yamamoto K, Oshima T, Nies DH, Silver S (2007). Molecular Microbiology of Heavy Metals.

[21] Smirnova GV, Muzyka NG, Glukhovchenko MN, Oktyabrsky ON (2000). Effects of menadione and hydrogen peroxide on glutathione status in growing *Escherichia coli*. Free Radic. Biol. Med.

[22] Helbig K, Bleuel C, Krauss GJ, Nies DH (2008). Glutathione and transition-metal homeostasis in *Escherichia coli*. J. Bacteriol.

[23] Quig D (1998). Cysteine metabolism and metal toxicity. Alter. Med. Rev.

[24] Nies DH (1999). Microbial heavy-metal resistance. Appl. Microbiol. Biotechnol.

[25] Turner RJ, Weiner JH, Taylor DE (1998). Selenium metabolism in *Escherichia coli*. Biometals.

[26] Stocker J, Balluch D, Gsell M, Harms H, Feliciano J, Daunert S, Malik KA, van der Meer JR (2003). Development of a set of simple bacterial biosensors for quantitative and rapid measurements of arsenite and arsenate in potable water. Environ. Sci. Technol.

[27] Wright DA, Dawson R, Cutler SJ, Cutler HG, Orano-Dawson CE, Graneli E (2007). Naphthoquinones as broad spectrum biocides for treatment of ship's ballast water: Toxicity to phytoplankton and bacteria. Water Res.

[28] Stohs SJ, Ohia S, Bagchi D (2002). Naphthalene toxicity and antioxidant nutrients. Toxicology.

[29] Wilson AS, Davis CD, Williams DP, Buckpitt AR, Pirmohamed M, Park BK (1996). Characterisation of the toxic metabolite(s) of naphthalene. Toxicology.

[30] Koutsaftis A, Aoyama I (2006). The interactive effects of binary mixtures of three antifouling biocides and three heavy metals against the marine algae *Chaetoceros gracilis*. Environ. Toxicol.

[31] Mochida K, Fujii K, Arai T, Harino H, Ohji M, Langston WJ (2009). Toxicity in plankton and fish Kazuhiko Mochida and Kazunori Fujii. Ecotoxicology of Antifouling Biocides.

[32] Appel KE (2004). Organotin compounds: Toxicokinetic aspects. Drug. Metab. Rev.

[33] Zazueta C, Reyes-Rivas H, Bravo C, Pichardo J, Corona N, Chávez E (1994). Triphenyltin as inductor of mitochondrial membrane permeability transition. J. Bioenerg. Biomembr.

[34] Robertson JD, Orrenius S (2000). Molecular mechanisms of apoptosis induced by cytotoxic chemicals. Crit. Rev. Toxicol.

[35] Ellman GL (1959). Tissue sulfhydryl groups. Arch. Biochem. Biophys.

[36] Inayat-Hussain SH, Chan KM, Rajab NF, Din LB, Chow SC, Kizilors A, Farzaneh F, Williams GT (2010). Goniothalamin-induced oxidative stress, DNA damage and apoptosis via caspase-2 independent and Bcl-2 independent pathways in Jurkat T-cells. Toxicol. Lett.

